# The length of susceptibility vessel sign predicts early neurological deterioration in minor acute ischemic stroke with large vessel occlusion

**DOI:** 10.1186/s12883-021-02455-7

**Published:** 2021-10-29

**Authors:** Lanying He, Jian Wang, Feng Wang, Lili Zhang, Lijuan Zhang, Wang Zhao, Xiechuan Weng, Fan Xu

**Affiliations:** 1 Department of Neurology, The Second People’s Hospital of Chengdu, Chengdu, 610021 People’s Republic of China; 2 Department of Neurology, The Second Affiliated Hospital of Chengdu College, Nuclear Industry 416 Hospital, Chengdu, 610021 People’s Republic of China; 3 Department of Neurology, Yongchuan Hospital, Chongqing Medical University, Chongqing, 610020 People’s Republic of China; 4 Beijing Institute of Basic Medical Sciences, Beijing, 100850 China; 5 School of Public Health Chengdu Medical College, Chengdu, 610500 Sichuan China

**Keywords:** Acute minor stroke, Susceptibility vessel sign, Large vessel occlusion, Early neurological deterioration

## Abstract

**Background:**

Patients with acute large vessel occlusion (LVO) presenting with minor stroke are at risk of early neurological deterioration (END). The present study aimed to evaluate the frequency and potential predictors of END in patients with medical management and LVO presenting with minor stroke. The relationship between SVS length and END was also investigated.

**Methods:**

This was a prospective multicenter study. Consecutive patients were collected with anterior circulation. LVO presented with minor stroke [National Institutes of Health Stroke Scale (NIHSS) ≤ 4] within 24 h following onset. END was defined as a deterioration of NIHSS ≥4 within 24 h, without parenchymal hemorrhage. The length of the susceptibility vessel sign (SVS) was measured using the T2* gradient echo imaging.

**Results:**

A total of 134 consecutive patients with anterior circulation LVO presenting with minor stroke were included. A total of 27 (20.15%) patients experienced END following admission. Patients with END exhibited longer SVS and higher baseline glucose levels compared with subjects lacking END (*P* < 0.05). ROC curve analysis indicated that the optimal cutoff point SVS length for END was SVS ≥ 9.45 mm. Multivariable analysis indicated that longer SVS [adjusted odds ratio (aOR), 2.03; 95% confidence interval (CI), 1.45–2.84; *P* < 0.001] and higher baseline glucose (aOR,1.02; 95% CI, 1.01–1.03; *P* = 0.009) levels were associated with increased risk of END. When SVS ≥ 9.45 mm was used in the multivariate logistic regression, SVS ≥ 9.45 mm (aOR, 5.41; 95%CI, 1.00–29.27; *P* = 0.001) and higher baseline glucose [aOR1.01; 95%CI, 1.00–1.03; *P* = 0.021] were associated with increased risk of END.

**Conclusions:**

END was frequent in the minor stroke patients with large vessel occlusion, whereas longer SVS and higher baseline glucose were associated with increased risk of END. SVS ≥ 9.45 mm was a powerful independent predictor of END.

## Background

Patients with minor neurological symptoms of acute ischemic stroke (AIS) are frequent. Neurological symptoms account for approximately half of all patients with AIS [1, 2]. Although minor strokes are considered benign, nearly one-third of the subjects affected have a persistent disability [3–5]. Previous studies have shown that approximately 30% of minor strokes are caused by acute large vessel occlusion (LVO). The affected patients were at high risk of early neurological deterioration (END) [6, 7].

The susceptibility vessel sign (SVS) on gradient echo (GRE) imaging is defined as a hypointense signal exceeding the diameter of the contralateral artery at the site of the thrombus [8, 9]. The SVS is related to the presence of deoxyhemoglobin, which causes inhomogeneities in local magnetic fields and therefore signal loss on the T2* sequences. Certain studies have suggested that SVS is associated with cardioembolic (CE) subtype [10–12] and the characteristics of SVS, such as their diameter and length, may predict stroke subtypes [13].

Recent studies have shown that SVS is significantly associated with lower early [14] or 24-h [13, 15] recanalization rate in the AIS group with intravenous thrombolysis [13–15]. Longer thrombus size was independently associated with END in patients with minor stroke and LVO following intravenous thrombolysis [16].

Accurate prediction of END in patients with minor stroke and large vessel occlusion following medical treatment may aid clinicians to select appropriate treatment, such as thrombectomy.

However, the relationship between SVS length and END in patients with minor AIS and LVO receiving medical treatment has been studied to a lesser extent. Therefore, the current study aimed to evaluate the frequency and potential predictors of END in medically managed patients with LVO presenting with minor AIS. Moreover, the relationship between SVS length and END was investigated.

## Methods

### Study population

The present study was a multicenter prospective study conducted in the three following medical centers: The Second People’s Hospital of Chengdu, Yongchuan Hospital of Chongqing Medical University and the Nuclear Industry 416 Hospital. The patients with AIS were admitted within 24 h of the onset symptoms between May 2014 and September 2020, who were diagnosed as AIS according to the WHO diagnostic criteria and were confirmed with the brain computed tomography (CT) or diffusion-weighted imaging (DWI) using Siemens Magnetom Avan to 1.5 Tesla (Siemens Medical Solutions, Erlangen, Germany) on admission. LVO was confirmed with computed tomography angiography (CTA) or magnetic resonance angiography (MRA). The initial severity of stroke was assessed by the National Institutes of Health Stroke Scale (NIHSS).

### Inclusion and exclusion criteria

The patients were enrolled only if they fulfilled the following criteria: 1. Age ≥ 18 years; 2. Admission for first-ever acute ischemic stroke within 24 h; 3. NIHSS≤4; 4.Evidence for ischemic lesions consistent with clinical presentation; 5. Acute LVO in the anterior circulation (defined by occlusion of the intracranial internal carotid artery, proximal segment of the middle cerebral artery [M1 or M2 trunk], or tandem occlusion). 6. All patients received standard therapy (including antiplatelet agents, statins, blood pressure control, or intravenous thrombolysis). The exclusion criteria were the following: 1. Ongoing infection at admission; 2. A history of previous stroke; 3. Moyamoya; 4. Treatment with endovascular therapy (EVT) prior to END; 5. Cerebral hemorrhage, hypoxia (arterial oxyhemoglobin saturation < 90%); 6. Contraindications for MRI examination.

### Clinical assessment and definition of END

The characteristics and vascular risk factors of the patients were collected. Blood cell counts, lipid and glucose levels were obtained on admission.

END was defined as neurological symptoms with an increase in the total NIHSS ≥4 that was attributed to the underlying LVO (excluding the subjects related to hemorrhagic transformation or medical complications) within 24 h.

MR imaging was performed on a 1.5 T magnet (Siemens Medical Solutions, Erlangen, Germany) with a phased array head coil. The acquisition parameters for GRE T2* were the following: TR, 800 ms; TE, 30 ms; section thickness, 5 mm; intersection gap, 1 mm; FOV, 250 × 250 mm; flip angle, 20°. All patients were imaged on the same 1.5-T MRI system and the same parameters for GRE T2 were used in three medical centers.

**Fig. 1:**
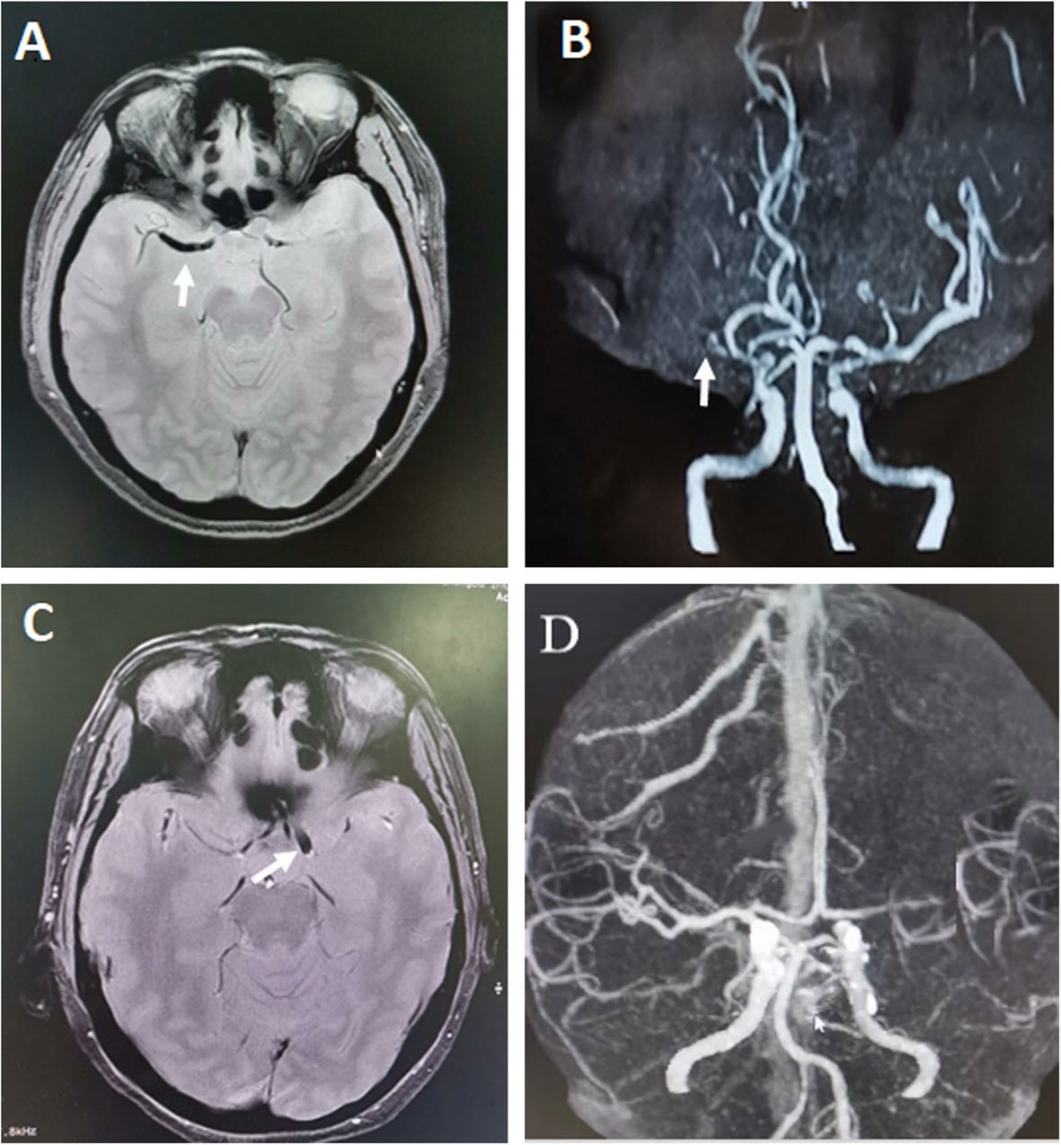
Examples of long SVS and short SVS. MRI of 2 patients presenting with long SVS (**A** and **B**, white arrow) and short SVS (**C** and **D**, white arrow); **A** and **C**, axial views of T2*-GRE; **B**, MRA; **D**, CTA

SVS length was measured and its location was noted according to the predefined arterial segments. The in-plane length of the clot corresponded to the distance between the proximal and distal part of the SVS; the length of the clot perpendicular to the axial acquisition plane was obtained by multiplying the number of cross-sectional locations where the clot was visible by the slice thickness (Fig. 1).

All generated images were stored on a CD-ROM with the visualization software (Siemens Germany). Two radiologists independently reviewed all MRI images without the clinical data and potential disagreements were resolved by discussion.

### Statistical analysis

Firstly, patients were classified into no END and END groups. The data are presented as median values (interquartile range [IQR]), numbers (%), or mean values (±standard deviation). To identify the differences between subgroups, the Pearson χ^2^ test was used for categorical variables. The distributions of the continuous variables were determined by the Kolmogorov–Smirnov test, while the Mann–Whitney two sample test was applied in case of non-normal distributions. Secondly, receiver operating characteristic (ROC) curve analysis was used to evaluate sensitivity, specificity and to determine the optimal cutoff point of length of SVS for END. Thirdly, the variables associated with END in the univariate analyses with a *P*-value < 0.20 were included in the multivariate analysis. Subsequently logistic regressions analyses were performed to determine the association between specific factors and END. The results were expressed as adjusted odds ratios (aOR) with the corresponding 95% confidence interval (CI). The data were analyzed using SPSS software (SPASS 22.0). *P* < 0.05 was considered to indicate statistically significant differences.

## Results

### Characteristics of the study subjects

A total of 174 patients with acute LVO presenting with minor stroke were enrolled at the three centers. Among them, 2 patients demonstrated infection at admission, whereas 17 patients exhibited a history of previous stroke, 1 had Moyamoya disease and 3 had cerebral hemorrhage transformation. A total of 11 subjects had contraindications for MRI examination, while 6 patients were directly transferred for EVT. The remaining 134 patients were recruited. The mean age was 67.01 ± 10.15 years (45.8–96.8 years) and the sample size comprised 50% (67) men. In the study population, 111 patients had a history of hypertension, 51 presented with a history of diabetes, 90 exhibited a history of hyperlipidemia, 24 were current smokers and 33 were current alcohol drinkers. The occlusion sites were intracranial ICA in 34 patients (25.37%), MCA M1 in 65 patients (48.51%) and MCA M2 in 35 patients (26.12%). The median NIHSS score at admission was 2 (interquartile range, 1–3). A total of 35 (26.12%) patients underwent intravenous thrombolysis. Among them, 27 (20.15%) patients experienced END following admission.

### Evaluation of the prognostic value of SVS for END

**Fig. 2:**
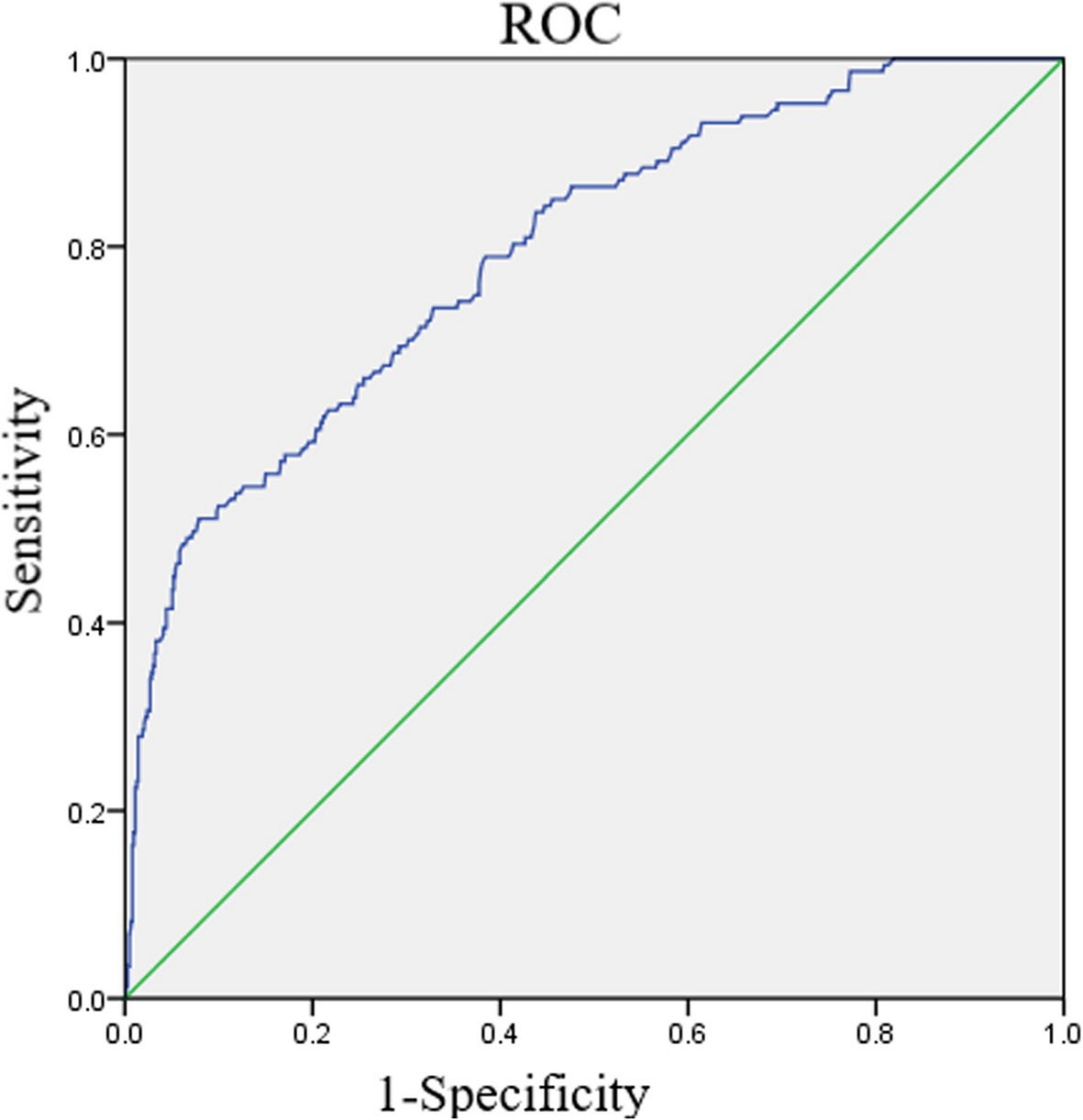
Receiver operating characteristic curve analysis for SVS. The curve was used for determining the prognostic value of END

ROC curve analysis indicated high accuracy for SVS in order to predict END with AUC of 0.74 (95% CI 0.63 to 0.84) (Fig. 2). By using a cutoff value of SVS ≥ 9.45 mm, the sensitivity and specificity was 77.78 and 56.07%, respectively, which provided a positive predictive value (PPV) for END of 30.89% and a negative predictive value (NPV) of 90.91%.

### Univariable models for predictors of END

A total of 27 (20.15%) patients experienced END following admission. Patients with SVS ≥ 9.45 mm experienced more END [30.88% (21/68) versus 9.09% (6/66), *P* = 0.002]. The baseline characteristics of patients in the no END and END groups were compared (Table 1). At baseline, patients with END indicated significantly higher baseline glucose levels (156.88 ± 43.67 vs. 128.75 ± 39.95, *P* < 0.001), and longer SVS [10.68(9.70–13.25) vs. 9.13(7.73–10.41), *P* < 0.001] than patients with no END. No significant differences were noted in the occlusion site between the groups with or without END (*P* > 0.05).

**Table 1:** Comparison of baseline characteristics between patients with no END and END

	no END group (107)	END group (27)	OR(95%CI)	P*
Age, y (Mean SD)	66.75 ± 10.51	68.01 ± 8.66		**0.334**
NIHSS score, median (IQR)	2(1–3)	2(1–3)		**0.279**
SVS length, median (IQR)	9.13(7.73–10.41)	10.68(9.70–13.25)		**< 0.001**
SVS ≥ 9.45 mm, n(%)	47(43.93)	21(77.78)	4.47(1.67–11.96)	**0.002**
Time from last seen well to MRI, h (Mean SD)	10.5 ± 7.8	11.2 ± 7.2		0.591
Baseline glucose, mg/dl (Mean SD)	128.75 ± 39.95	156.88 ± 43.67		**< 0.001**
DWI-ASPECTS, (Mean SD)	8.98 ± 1.25	8.93 ± 1.41		**0.607**
Females, n(%)	54(50.46)	13(48.15)	0.91(0.39–2.12)	0.829
Men, n(%)	53(49.53)	14(51.85)	0.91(0.39–2.12)	0.829
BMI ≥ 24 kg/m, n(%)	27(25.23)	5(18.52)	0.67(0.23–1.95)	0.465
Hypertension, n(%)	91(85.06)	20(74.07)	0.50(0.18–1.38)	0.177
Current Smoking, n(%)	22(20.56)	2(7.41)	0.31 (0.07–1.41)	0.111
Current alcohol drinking, n(%)	27(25.23)	6(22.22)	0.85(0.31–2.32)	0.746
Diabetes, n(%)	42(39.25)	9(33.33)	0.77(0.32–1.88)	0.571
Hyperlipidemia, n(%)	73(68.22)	17(62.96)	0.79(0.33–1. 91)	0.603
Thrombolytic therapy, n(%)	29(27.10)	6(22.22)	0.77(0.28–2.09)	0.606
Family history of stroke, n(%)	14(13.08)	5(18.52)	1.51(0.49–4.64)	0.469
Occlusion site				
Intracranial ICA, n (%)	26(24.30)	8(29.63)	1.31(0.51–3.35)	0.569
MCA M1, n (%)	52(48.60)	13(48.18)	0.98(0.42–2.29)	0.967
MCA M2, n (%)	29(27.10)	6(22.22)	0.77(0.28–2.09)	0.606
TOAST				
Cardiac embolism	22(20.56)	9(33.33)	1.93(0.76–4.88)	0.160
LAA, n (%)	30(28.04)	7(25.93)	0.90(0.34–2.34)	0.826
Lacunar, n (%)	30(28.04)	8(29.63)	1.08(0.43–2.73)	0.870
Undetermined, n (%)	23(21.50)	2(7.40)	0.29(0.064–1.33)	0.093
Other, n (%)	2(1.87)	1(3.70)	2.02(0.18–23.13)	0.565
Medications use				
Antiplatelet, n(%)	29(27.10)	6(22.22)	0.77(0.28–2.01)	0.606
Antihypertensive, n(%)	60(56.07)	14(51.85)	0.84(0.36–1.97)	0.693
Lipid-lowering medications, n(%)	54(50.47)	13(48.15)	0.91(0.39–2.12)	0.829

Bold indicates *P*-values less than 0.05

*Comparison between no END and END groups. The data are presented as median values (interquartile range [IQR]), numbers (%), or mean values (±standard deviation). Categorical variables are expressed as frequency (percent) for *P* values. Continuous variables are expressed as mean ± standard deviation (SD). Baseline characteristics were compared between the 2 subgroups by univariate analysis using Pearson χ^2^ and the distributions of continuous variables were determined by the Kolmogorov–Smirnov test. The Mann–Whitney two sample test was applied in the case of non-normal distributions

### Multivariable models on the association between SVS ≥ 9.45 mm and END

In the unadjusted models, an association between SVS ≥ 9.45 mm and END was noted (OR, 4.66; 95%CI, 3.24–6.72, *P* < 0.001). When the factors were associated with END in the univariate analyses (*P* < 0.20) they were used into the multivariate logistic regression analysis (adjustment for SVS value, baseline glucose, hypertension, current smoking, cardiac embolism, undetermined stroke). The results indicated that longer SVS [aOR, 2.03; 95%CI, 1.45–2.84; *P* < 0.001] and higher baseline glucose (aOR, 1.02; 95% CI, 1.01–1.03; *P* = 0.009) were associated with increased risk of END. When SVS **≥** 9.45 mm was used in the multivariate logistic regression, (aOR, 5.41; 95%CI, 1.00–29.27; *P* = 0.001), higher baseline glucose levels (aOR1.01; 95%CI, 1.00–1.03; *P* = 0.021] were associated with increased risk of END (Table 2).

**Table 2:** Multivariable models showing predictors of END

	aOR (95% CI)	P*
Model 1 (SVS length)		
SVS length	2.03(1.45–2.84)	**< 0.001**
Baseline glucose	1.02(1.01–1.03)	**0.009**
Model 2 (SVS ≥ 9.45 mm)		
SVS ≥ 9.45 mm	5.41(1.00–29.27)	**0.001**
Baseline glucose	1.01(1.00–1.03)	**0.021**

Bold indicates *P*-values less than 0.05

*Multivariable adjusted for baseline glucose, hypertension, current smoking, cardiac embolism, undetermined stroke, SVS value or SVS ≥ 9.45 mm

## Discussion

The present study indicated that 20.15% (27) of patients experienced END in minor stroke with LVO. The optimal length of SVS for predicting END was SVS ≥ 9.45 mm. Furthermore, we found that higher baseline glucose levels and longer SVS were associated with increased risk of END in patients with minor stroke and LVO.

Various acute AIS patients experience neurological deterioration during hospitalization. The common feature for neurological deterioration in these patients is the presence of an underlying LVO [17–20]. According to previous studies, END incidence ranged from 12 to 40% in patients with minor AIS and LVO [16, 21, 22]. A recent study indicated that the incidence of END in mild stroke patients (NIHSS≤5) with LVO was 19.7% [22], whereas another study reported that END occurred in 39.4% in minor stroke and LVO [21]. In a previous study conducted in France, the results indicated that END occurred in 12.1% patients with minor stroke and LVO was intended for intravenous thrombolysis alone [16]. These differences may reflect the clinical environment following stroke, including the inconsistent definitions of neurological deterioration, the medical conditions in the hospital, the differences in nursing conditions and the admission of the patients to the stroke unit. Our findings are in line with the results reported from previously published studies [16, 21–23].

A previous study indicated that the site of occlusion was the predictor of the risk of END, which was reported to be more frequent in the carotid terminus and in the tandem occlusions [23]. However, the applicability of these findings to LVO strokes is questionable since these studies included patients with no LVO stroke, patients with moderate to severe stroke, or exhibited small sample size and inconsistent definitions of END. No statistical differences were noted in the occlusion site between the groups with or without END. These differences may be due to the inclusion of patients with more proximal anterior circulation occlusions (intracranial internal carotid artery, middle cerebral artery M1/2, or tandem occlusion) in the current study. Based on magnetic resonance imaging (MRI) and T2*-gradient echo imaging sequence (T2*-gradient echo imaging), the thrombus with high red blood cell content exhibited low signals, which is termed susceptible vessel sign (SVS)+. SVS is related to the presence of deoxyhemoglobin, which leads to the inhomogeneity of local magnetic field and the loss of the T2* signal. Previous studies indicated that decreased SVS length was associated with increased recanalization rate [24–26]. In the present study, we aimed to investigate the association between SVS length and END in patients with minor stroke and LVO. The data demonstrated that longer SVS was associated with increased risk of END in patients with minor stroke and LVO. The END group exhibited a significantly higher SVS than that of the non-END group. The results were consistent with those reported in previous studies [16].

Although the exact mechanisms of END are unclear, one hypothesis is that the symptomatic ischemic tissue (i.e. infarct core and/or penumbra) is extended into the surrounding asymptomatic tissue [27–29]. Only emboli containing deoxyhemoglobin or hemosiderin can be identified as SVS. In other words, SVS is a predictor of older thrombosis. Longer SVS represent a higher clot burden, which is associated with decreased effectiveness of medical treatment and leads to secondary hemodynamic and metabolic disorders. It may be the mechanism of END in patients with minor stroke presented with LVO.

In a previous study, it was shown that thrombus length>9 mm was a powerful predictor of lack of early recanalization following intravenous thrombolysis [14]. In the present study, the optimal cutoff point of SVS length for END was SVS ≥ 9.45 mm. However, following adjustment for confounders, a significant association of SVS ≥ 9.45 mm was noted with risks of END in patients with minor stroke and LVO. Therefore, longer thrombus length (especially> 9 mm) was associated with poor prognosis [14, 16]. The results of the present study indicated that the length of SVS was a valuable marker of END in patients following minor stroke.

It has been shown that patients with hyperglycemia exhibit increased risk for stroke and notably ischemic stroke [30]. Elevated levels of blood glucose in the peri-infarct period are closely associated with poor outcome in patients with AIS [31–34]. In patients with END, blood glucose levels were significantly elevated and the possible mechanisms associated with this outcome included poor collateral flow and endothelial cell injury, oxidative stress and lactic acid increase, glucose and energy metabolism disorders. In the present study, higher baseline glucose levels were associated with increased risk of END, which was consistent with previous studies [31–34].

Given the correlation between END and SVS length in patients with mild stroke with LVO, restoration of perfusion is considered a potential treatment strategy for patients at high risk of END. EVT may be a treatment option for patients with long SVS. However, further clinical trials should be designed to demonstrate the benefit of immediate EVT in patients with minor stroke and LVO who are at high risk for END.

The present study contains certain limitations that merit consideration. Firstly, in the present study, data on stroke volume were not available. Secondly, we did not analyze the perfusion images, which may influence the results. Thirdly, SVS was detected using MRI, which required longer time than computed tomography in LVO. MRI was not available for certain stroke patients. However, despite these limitations, the present study was a multicenter clinical study, which required the collection of several important clinical data and the adjustment of data analyses for a wide variety of confounding factors. The results of this study were useful for clinical treatment.

## Conclusions

In conclusion, the present study indicated that END was frequent in minor stroke patients with LVO. Longer SVS and that higher baseline glucose levels were associated with increased risk of END. Moreover, SVS ≥ 9.45 mm was a powerful independent predictor of END. The END prediction is very important, which can reduce mortality and improve the outcome. Length SVS may have potential predictive value in risk stratification of ischemic stroke, which may help to select high-risk patients to initiate intervention in time. The results of the present study imply that this method may be applicable in AIS management.

## Data Availability

The datasets used and/or analyzed during the current study are available from the corresponding author on reasonable request.

## Abbreviations

CI: Confidence Interval
M: Mean
OR: Odds Ratio
SD: Standard Deviation
END: Early neurological deterioration
LVO: Large vessel occlusion
NIHSS: National Institutes of Health Stroke Scale
SVS: Susceptibility vessel sign (SVS)
IQR: Interquartile range
LAA: Large-artery atherothrombosis
TOAST: Trial of Org 10,172 in Acute Stroke Treatment
EVT: Endovascular therapy
IVT: Intravenous thrombolysis
